# Appropriate Dosing Regimens of Non-Vitamin K Antagonist Oral Anticoagulants for Treatment of Patients With Non-Valvular Atrial Fibrillation: An Evidence-Based Consideration

**DOI:** 10.3389/fphar.2020.01293

**Published:** 2020-08-20

**Authors:** Shujuan Zhao, Xuejiao Hong, Jingjing Cao, Haixia Cai, Song Du, Peizhi Ma

**Affiliations:** ^1^ Department of Pharmacy, Henan Provincial People’s Hospital, People’s Hospital of Zhengzhou University, School of Clinical Medicine, Henan University, Zhengzhou, China; ^2^ Department of Cardiovascular Medicine, Henan Provincial People’s Hospital, People’s Hospital of Zhengzhou University, School of Clinical Medicine, Henan University, Zhengzhou, China

**Keywords:** stroke prevention, non-valvular atrial fibrillation, non-vitamin K antagonist oral anticoagulants, clinical settings, dosing regimens

## Abstract

Patients with non-valvular atrial fibrillation (NVAF) exhibit a high risk of stroke, which is associated with high mortality. Thus, stroke prevention is crucial for the overall management of NVAF. Two categories of drugs, vitamin K antagonist warfarin and non-vitamin K antagonist oral anticoagulants (NOACs), are clinically used to prevent NVAF-related stroke. In some circumstances, NOACs are superior to warfarin. However, NOACs selection for NVAF patients is affected by many factors, including individual patient characteristics, comorbidities, risk factors, or laboratory variables. This article summarizes the discrepancy in NOACs management with emphasis on the dosing regimens and influencing factors, such as stroke risk, age, body weight, renal function, gastrointestinal bleeding (GIB) risk, and combination of antiplatelet therapy, in order to identify individual groups with particular clinical characteristics who may obtain more benefit from a certain dosing regimen of NOACs. Determination of a particular subset of patient populations for the appropriate dose regimen of NOACs will help to achieve desired clinical outcomes. Furthermore, to compensate clinical evidence, we should place more emphasis on the findings of current clinical trials and supplement real-world data.

## Introduction

Atrial fibrillation (AF) is the most common heart arrhythmia and is linked to an elevated risk of systemic embolism (SE) and ischemic stroke (IS) (Camm et al., 2012). Oral anticoagulation has been shown to reduce IS and SE by more than 60% and decrease the mortality risk in AF patients (Potpara et al., 2019). Vitamin K antagonists (VKAs), such as warfarin, are effective in the prevention of AF-related stroke. However, clinical management of VKAs is difficult because of their narrow therapeutic index, required frequent laboratory monitoring, and drug and diet interactions (Zhao et al., 2019). Alternatively, direct oral anticoagulants (DOACs), also known as non-vitamin K antagonist oral anticoagulants (NOACs), have been developed (Zhao et al., 2019).

NOACs include dabigatran (factor IIa inhibitor), rivaroxaban, apixaban, edoxaban, and betrixaban (factor Xa inhibitors) (**Supplementary Material, Figure S1**). Phase III randomized controlled trials (RCTs) showed that NOACs are at least non-inferior to warfarin in terms of IS/SE prevention and have a low rate of intracranial hemorrhage (ICH) and major bleeding events for stroke prevention in atrial fibrillation (SPAF) (Connolly et al., 2009; Granger et al., 2011; Patel et al., 2011; Giugliano et al., 2013). Hence, NOACs are strongly recommended by the current guidelines as a substitute for warfarin in patients with non-valvular atrial fibrillation (NVAF) (Kirchhof et al., 2016; January et al., 2019).

NOACs have been marketed extensively globally, however, the appropriate use of NOACs is a considerable issue. An observational study (Yao et al., 2017) examined the standard doses for participants with a renal indication for both potential over-dosing and under-dosing of NOACs, and showed that prescribed NOAC doses were often not in accordance with drug labeling, which may be linked to poor safety and no benefit in those patients with severe kidney disorders. Another study in Korea (Jung et al., 2018) investigated the distinction in stroke outcomes in NVAF patients based on their previous medication status, including under-dosed versus standard-dosed NOACs. Among 858 patients examined, standard-dosed NOACs or warfarin with treatment intensity was linked to a comparatively mild stroke in NVAF patients (Jung et al., 2018).

Owing to the clinical heterogeneity of AF patients, few studies have sought to uncover whether the appropriate patients are being treated and whether specific patient populations are receiving correct NOACs doses. In this article, we will outline the dosage suggestions for NVAF patients in different nations, and then provide a general outline on the NOACs performance in AF patients with particular clinical characteristics. We will reflect on how dose adjustment should be recommended based on the current knowledge, with the goal of presenting a simple and workable strategy for clinicians to choose an appropriate NOAC.

## Dosing Regimens Perspectives and Supporting Data

Apart from betrixaban, other NOACs have been examined for SPAF in patients during confirmatory phase III global RCTs (e.g. ENGAGE AF-TIMI with edoxaban, ROCKET-AF with rivaroxaban, RE-LY with dabigatran, and ARISTOTLE with apixaban) and were proven to have favorable safety and efficacy (Connolly et al., 2009; Granger et al., 2011; Patel et al., 2011; Giugliano et al., 2013). However, different doses of these agents were evaluated differently (Steffel et al., 2018). In ENGAGE AF-TIMI (edoxaban) and RE-LY (dabigatran) (Connolly et al., 2009; Giugliano et al., 2013), no pre-defined dose-decrease criteria were set; either a higher or a lower dose was verified in the fully powered cohorts (dose-reducing for edoxaban in particular patients). In contrast, in ARISTOTLE (apixaban) and ROCKET-AF (rivaroxaban) (Granger et al., 2011; Patel et al., 2011), the dose was decreased under the circumstance of pre-defined patient characteristics. For rivaroxaban and edoxaban, a standard dose was reduced in patients with one specific risk factor; however, for apixaban, data supported the dose adjustment to 2.5 mg twice daily when a patient had two of three characteristics (age ≥ 80 years, serum creatinine ≥ 1.5 mg/dL, or weight ≤ 60 kg) (**Table 1**). In addition, different countries do not adopt the same rules of NOACs prescription, and local policies, like formulary committees, regulatory approval, and cost-effectiveness, all influence NOACs availability (**Supplementary Material, Table S1**). Therefore, to reproduce the positive results from the RCTs, it is critical to use the correct dose regimen.

**Table 1:** NOACs and studied doses in SPAF.

NOACs	Clinical trial	Standard dose	Dose reduction
Dabigatran	RE-LY	2 × 110 mg 2 × 150 mg	No pre-specified dose-reduction criteria. 2 × 110 mg if: -age ≥80 years; -elevated risk of gastrointestinal bleeding; -concomitant verapamil;
Rivaroxaban	ROCKET-AF	1 × 20 mg	1 × 15 mg if: -CrCl ≤50 ml/min
Apixaban	ARISTOTLE	2 × 5 mg	2 × 2.5 mg if two out of three: -age ≥80 years -weight ≤60 kg; -serum creatinine ≥133 μmol/L (1.5 mg/dl) [or if CrCl 15–29 ml/min];
Edoxaban	ENGAGE AF-TIMI	1 × 60 mg	1 × 30 mg if: -CrCl ≤50 ml/min; -weight ≤60 kg; -concomitant use with strong P-gp inhibitor (verapamil, quinidine, or dronedarone);

NOAC, non-vitamin K antagonist oral anticoagulants; SPAF, stroke prevention in atrial fibrillation; CrCl, creatinine clearance; P-gp, P-glycoprotein.

The data summarized in this table are from (Steffel et al., 2018).

According to the results obtained from the RE-LY trial, two dosages of dabigatran, 150 and 110 mg, exhibited superior or non-inferior efficacy and safety compared to warfarin (Connolly et al., 2009); however, various countries have made different approvals for dabigatran dosing. Because the 150 mg dose was more effective at significantly reducing the stroke occurrence rate compared to the 110 mg dose, the US Food and Drug Administration (FDA) only approved the 150 mg dose, even though the larger dose was equal to warfarin with regards to risk of major bleeding events (Beasley et al., 2011; Cho et al., 2019). To balance the concerns about a lack of a low dose option, the FDA approved a 75 mg dose regimen of dabigatran based on pharmacokinetic simulations instead of efficacy and safety data to treat severe renal impaired patients (Steffel et al., 2018). However, the 75 mg regimen has not been tested in any clinical trial and additional RCTs are therefore necessary. By contrast, the European Medicines Agency (EMA) and the Chinese National Medical Products Administration (NMDA) focused on the bleeding risk of dabigatran and not only endorsed the 150 mg dose, but also recommended the 110 mg dose as a decreased-dose for frail patients (aging, concomitant verapamil, or other increased breeding risk) (Diener et al., 2017b; Steffel et al., 2018). It should be noted that the 75 mg regimen has not been approved for SPAF by the EMA and NMDA.

The FDA and EMA (but not the NMDA) approved the SPAF indication for apixaban. The efficacy and safety of apixaban for the above indications were instituted by the AVERROES and ARISTOTLE trials (Connolly et al., 2011; Granger et al., 2011). Since apixaban has not been studied in populations in the Chinese mainland, the NMDA did not approve the SPAF indication for apixaban, which has limited the development and application of apixaban in China.

## Discussion

In the real world, it is not easy to fully replace NOACs despite the challenges in particular patient cohorts. Because of the clinical heterogeneity of NVAF patients and various clinical features that can change NOACs plasma concentrations, the appraisal of bleeding and stroke rapidly goes beyond the level of detail embodied on the labels (Desmaele et al., 2016). This process may be complicated, especially when a particular clinical profile or multiple risk factors are present, and physicians should make an appropriate decision based on guidelines, evidence-based studies, and risk optimization tools.

### Risk of Stroke

Large randomized prospective trials have shown that all NOACs treatments reduced ICH compared to well-controlled VKAs (Connolly et al., 2009; Granger et al., 2011; Patel et al., 2011; Giugliano et al., 2013). A meta-analysis analyzed the findings from the pivotal phase III AF RCTs and showed that NOACs were associated with a lower risk of stroke or SE (~19%) compared to warfarin, mainly due to significant decreases in hemorrhagic stroke and intracranial bleeding (ICB) (Ruff et al., 2014). The ENGAGE AF-TIMI 48 trial illustrated the influence of under-dosing (Giugliano et al., 2013). The Edoxaban 30/15 mg group presented a higher IS rate compared to the well-controlled VKA group, which led to disapproval of this dosing regimen for clinical application. In contrast, in the RE-LY study (Connolly et al., 2009), patients receiving 150 mg dabigatran had significantly reduced IS/SE rates compared to those receiving warfarin, and this higher dose was thus recommended for related clinical application. As with 110 mg of dabigatran and rivaroxaban, apixaban, and 60 mg of edoxaban, the stroke risk was comparable to that of warfarin (Connolly et al., 2009; Granger et al., 2011; Patel et al., 2011; Giugliano et al., 2013).

### Elderly

AF incidence increases steadily during the last decades of one’s lifespan (Wolff et al., 2015). Given that the stroke risk rises dramatically with age, anticoagulants provide net clinical benefit for older patients. In the phase III NVAF clinical trials (Connolly et al., 2009; Granger et al., 2011; Patel et al., 2011; Giugliano et al., 2013), some distinctions in bleeding risk rates for patients aged ≥ 75 years using different anticoagulants were observed (**Supplementary Material, Figure S2**). For individuals aged ≥ 80 years, a reduction of dabigatran dose to 110 mg was needed. For apixaban, if two out of three risk factors existed (based on age, creatinine, or weight), a reduced dose to 2.5 mg twice daily was recommended. No age-based dose adjustments for rivaroxaban or edoxaban have been recommended (**Table 1**). The EMA recommended dabigatran at 110 mg for patients aged ≥ 75 years with stroke risk, rather than the recommended 110 mg dose from the manufacturer in all NVAF patients aged ≥ 80 years. One previous study of NOACs used in elderly patients indicated a correlation between age and higher extracranial major bleeding with two dabigatran doses (Eikelboom et al., 2011). Conversely, a similar extracranial major bleeding rate was observed with edoxaban, rivaroxaban, and apixaban, independent of age (Halperin et al., 2014; Halvorsen et al., 2014; Kato et al., 2016). The ongoing ELDERCARE-AF study (Okumura et al., 2017) compares the safety and efficacy of edoxaban 15 mg once-daily versus placebo in Japanese NVAF patients aged ≥ 80 years who are not eligible for standard treatment of oral anticoagulation.

### Body Weight

Low body weight can increase NOACs exposure and risk of over-dosing (Braekkan et al., 2016). Of note, patients who have a low body weight commonly have other conditions, such as frailty, reduced muscle mass, cancer, and renal impairment, that may add to the risk of bleeding and stroke (Steffel et al., 2018). For patients with low body weight (< 50 kg), a daily dose of 300 or 220 mg dabigatran can be chosen according to the patient’s circumstance; dabigatran may be a less preferable option for an under-weight older AF patient with co-existing renal impairment (Undas et al., 2020). Body weight ≤ 60 kg is a dose-decreasing criterion for edoxaban as well as apixaban (if another risk factor is present). Since the efficacy and safety of edoxaban and apixaban are at least comparable with warfarin in underweight patients (Granger et al., 2011; Giugliano et al., 2013), either should be an appropriate choice for patients weighing < 60 kg. Body weight < 50 kg or > 120 kg only slightly affects plasma concentrations of rivaroxaban (less than 25%), and therefore dosing adjustment is not necessary.

Surprisingly, body weight was not one of the exclusion criteria in any of the NOACs trials for AF patients. Given limited data in extreme body weight, the International Society on Thrombosis and Haemostasis (ISTH) recommended that VKAs be considered for patients with a body weight > 120 kg or a body mass index (BMI) ≥ 40 kg/m^2^ (Martin et al., 2016). The European Heart Rhythm Association (EHRA) recommended assessing plasma levels if a patient who is receiving NOACs has a body weight < 50 kg or > 120 kg (Steffel et al., 2018).

### Renal Function

Patients with chronic kidney disease (CKD) have an elevated risk of NVAF, IS, and bleeding compared to individuals who have normal kidney function, and the risk further increases with the progression of CKD, especially among dialysis patients (Kalra et al., 2018). Four of the NOACs have varying degrees of renal clearance: 27% for apixaban, 35% for rivaroxaban, 50% for edoxaban, and 80% for dabigatran (Steffel et al., 2018). Thus, the renal function of patients on NOACs should be monitored at least annually. If the kidney functional impairment [i.e. creatinine clearance (CrCl) ≤ 60 ml/min] occurs, assessment should be more frequently executed (Steffel et al., 2018).

In the subgroup analyses of the pivotal phase III AF trials, the four NOACs presented reproducible efficacy and safety in patients who had mild to moderate CKD compared to non-CKD patients (Hijazi et al., 2014; Bohula et al., 2016; Fordyce et al., 2016; Hijazi et al., 2016). These findings suggest that NOACs can be used for patients with mild to moderate kidney dysfunction. Of note, the NOACs trials did not include patients with a CrCl of <30 ml/min (< 25 ml/min for apixaban in the ARISTOTLE trial) (Connolly et al., 2009; Granger et al., 2011; Patel et al., 2011; Giugliano et al., 2013). Currently approved labels allowing use of some NOACs for patients with a CrCl of 15 ml/min is supported by studies from the pharmacokinetic model. Low-dose regimens of rivaroxaban, edoxaban, and apixaban are recommended for patients with severe renal insufficiency (CrCl of 15–29 ml/min) (Turakhia et al., 2018; Jain and Reilly, 2019). In Europe and China, dabigatran (110 and 150 mg) should not be administered to AF patients who have severe kidney impairment (i.e. CrCl < 30 ml/min), whereas in the US, a lower-dose of dabigatran (75 mg) has been approved for AF patients with a CrCl of 15–30 ml/min (Steffel et al., 2018).

It should be noted that rivaroxaban, edoxaban, and apixaban have not been approved for hemodialysis patients in Europe and China, as these patients were excluded from the major clinical trials. However, in the US, apixaban was approved for application in hemodialysis patients in 2014 (Undas et al., 2020). In contrast, NOACs are considered for AF patients undergoing kidney transplantation. In this subset of patients, the dose regimen should be chosen based on the assessed kidney function, and potential drug–drug interactions between the concomitant immunosuppressive agents and NOACs should be taken into account (Steffel et al., 2018). Currently, two ongoing trials, RENAL-AF (NCT 02942407) and AXADIA (NCT 02933697), aim to illustrate the advantages of apixaban over VKAs in AF patients with end-stage kidney disease (ESKD) (Turakhia et al., 2018).

After reviewing the ENGAGE AF-TIMI 48 trial, the FDA concluded that there is potential for decreased efficacy of edoxaban (60 mg QD) among patients with a high CrCl (> 95 ml/min) compared to well-managed warfarin (Bohula et al., 2016). In view of this, the FDA issued a warning: “edoxaban should not be used in patients with a CrCl > 95 ml/min because of an increased IS risk compared to warfarin”, and recommended the application of other oral anticoagulation (OAC) agents in these patients^35^ (SAVAYSA, 2016). The NMDA and EMA also suggested that, “edoxaban should only be used in NVAF patients with a high CrCl after a careful evaluation of the individual thromboembolic and bleeding risk” (Steffel et al., 2018). A retrospective study of the ENGAGE AF-TIMI 48 data suggested that, although there was an evident reduction in the efficacy of edoxaban 60 mg QD, its net clinical benefit and safety were comparable with those of warfarin in AF patients who had various degrees of renal impairment (Bohula et al., 2016).

### Risk of Gastrointestinal Bleeding (GIB)

Several systematic reviews concluded that patients treated with NOACs had an increased GIB rate (Kovacs et al., 2015; Silverio et al., 2019). In patients at high-risk of GIB, VKA, or another NOAC other than dabigatran 150 mg, rivaroxaban 20 mg QD, or edoxaban 60 mg QD BID is preferable (**Supplementary Material, Figure S3**), as supported by the finding that the use of NOACs (especially full-dose rivaroxaban and dabigatran) was closely correlated with higher GIB events (Kirchhof et al., 2016). In RE-LY, dabigatran 110 mg BID was comparable to warfarin with regards to GIB risk, but dabigatran 150 mg BID was associated with increased GIB risk compared to warfarin (Connolly et al., 2009). In ROCKET AF, rivaroxaban 20 mg QD had a greater GIB annual risk than warfarin (Goodman et al., 2014), and administration of rivaroxaban to patients aged ≥75 years also significantly increased GIB risk compared to warfarin (Halperin et al., 2014). The ENGAGE AF-TIMI trial showed a higher GIB risk with edoxaban 60 mg QD than with warfarin (HR 1.23), and correspondingly, a low-dose edoxaban (30 mg QD) was linked to a less GIB risk (Giugliano et al., 2013). NOAC-linked GIB is potentially associated with the following considerations: 1) Anticoagulation can be local or systematic, and the existence of the active agent in the GI tract may promote bleeding from susceptible lesions. 2) NOACs may suppress GI mucosal healing. 3) Dabigatran or etexilate contains tartaric acid, which may induce direct caustic injury (Cheung and Leung, 2017). Although having a GIB history is not a contraindication for NOACs therapy, the existence of GI lesions, including GI ulceration, is contraindicated for administering NOACs.

### Combination of Antiplatelet Therapy

NVAF patients with stable coronary artery disease (CAD) or acute coronary syndrome (ACS) may need percutaneous coronary intervention (PCI) (Diener et al., 2017a). Since antiplatelet treatment is the key treatment for patients with ACS, CAD, or PCI, a combined therapy with anticoagulation is generally required for NVAF patients. In these patients with a high risk of complications, the risks of stent thrombosis, stroke, and bleeding (particularly intracranial hemorrhage) need to be considered. Stacking antithrombotic preparations (i.e. adding two anti-platelets to NOACs) will remarkably increase the bleeding risk, thus preventing the long-term triple therapy in daily practice (Steffel et al., 2018; January et al., 2019). To date, four prospective RCTs addressed the issue of OAC with ACS and/or undergoing PCI by comparing NOACs and warfarin in various combinations with antiplatelet agents (**Table 2**).

**Table 2:** Trial profiles for the four NOACs with ACS or PCI in atrial fibrillation^a^.

	Dabigatran(RE-DUAL PCI)	Rivaroxaban (PIONEER AF-PCI)		Apixaban (AUGUSTUS)	Edoxaban (ENTRUST AF-PCI)
Standard dose	150 mg twice daily or 110 mg twice daily	15 mg once daily	2.5 mg twice daily	5 mg twice daily	60 mg twice daily
Dose reduction in selected patients	No dose reduction	Rivaroxaban 10 mg once daily if CrCl 30–49 ml/min	No dose reduction	Apixaban 2.5 mg twice daily if at least two: age ≥80 years, body weight no more than 60 kg, serum creatinine ≥1.5 mg/dl (133 μmol/L)	Edoxaban 30 mg once daily if one or more factors were present: creatinine clearance of 15–50 ml/min, body weight ≤60 kg, concomitant use of specified potent P-glycoprotein inhibitors
P2Y12 inhibitor	P2Y12 inhibitor (clopidogrel or ticagrelor)	P2Y12 inhibitor (clopidogrel, prasugrel, or ticagrelor) for 12 months	Dual antiplatelet therapy (DAPT) for 1, 6, or 12 months	Planned to use a P2Y12 inhibitor (clopidogrel, prasugrel, or ticagrelor) for at least 6 months	P2Y12 inhibitor (clopidogrel, prasugrel, or ticagrelor) for 12 months
Study design	Randomized, open-label	Randomized, open-label		Randomized, two-by-two factorial, apixaban with VKA was open-label; aspirin with matching placebo was double-blind	Randomized, open-label
Number of patients	2,725	2,124		4,614	1,506

a As outlined in detail, the four clinical trials were designed to evaluate safety, but not designed to determine non-inferiority for efficacy endpoints.

NOAC, non-vitamin K antagonist oral anticoagulants; ACS, acute coronary syndrome; PCI, percutaneous coronary intervention; CrCl, creatinine clearance.

The data summarized in this table are from (Gibson et al., 2016; Cannon et al., 2017; Lopes et al., 2019; Vranckx et al., 2019).

In the PIONEER AF-PCI trial, two different dosing regimens containing rivaroxaban [rivaroxaban 15 mg with a P2Y12 inhibitor (rivaroxaban 10 mg in patients with a CrCl of 30–50 ml/min), or a P2Y12 inhibitor with rivaroxaban 2.5 mg twice daily and aspirin] were examined with standard triple therapy [VKAs and dual antiplatelet therapy (DAPT)] (Gibson et al., 2016). This trial suggested that both rivaroxaban regimens significantly decreased the rates of severe bleeding during a 1-year follow-up period compared with the standard triple treatment, although the sample size was too small to have a significant difference statistically.

In the RE-DUAL PCI trial, the safety of clopidogrel or ticagrelor (without aspirin) and two dosages of dabigatran (150 or 110 mg BID) were compared with standard triple therapy containing aspirin, VKA, and either ticagrelor or clopidogrel in NVAF patients undergoing PCI (Cannon et al., 2017). The RE-DUAL PCI trial was not designed for individual efficacy endpoints; instead, the goal of this trial was to show whether the combined dual-treatment arms were inferior to the triple treatment arm with regard to various endpoints including thromboembolic events, death, and unplanned revascularization. It showed that both doses of dabigatran substantially lowered the major and non-major bleeding events, and were superior (150 mg) or non-inferior (110 mg) to VKA for SPAF.

The AUGUSTUS trial was an international trial with a two-by-two factorial design (Lopes et al., 2019). A total of 4,614 NVAF patients with PCI or ACS who planned to take a P2Y12 inhibitor were administered apixaban or VKA, and received aspirin or a placebo for 6 months. The primary endpoint was major or clinically relevant non-major (CRNM) bleeding, and the secondary endpoints included hospitalization or death and a composite of ischemic events. This study indicated that an antithrombotic regimen (with apixaban, without aspirin) group had fewer hospitalizations and bleeding events, but had comparable ischemic events compared with the regimen groups including aspirin, VKA, or both. AUGUSTUS also showed that dual therapy strategies (clopidogrel plus NOACs) were safer than the dual therapy of clopidogrel plus VKA with regard to bleeding risk.

The ENTRUST AF-PCI study was designed to reveal appropriate dosing regimens of antithrombotic therapy for NVAF patients with PCI. These patients received edoxaban (60 mg once daily, or reduced to 30 mg per day based on CrCl, body weight, or P-gp inhibitors) plus a P2Y12 inhibitor for 12 months, or VKA combining a P2Y12 inhibitor and aspirin for 1–12 months (Vranckx et al., 2019). This trial revealed that for NVAF patients who had PCI, the edoxaban-based regimen was not inferior to the VKA-based regimen in terms of bleeding events and was comparable in terms of ischemic events.

According to the published RCTs, compared to the VKA regimen, the NOACs regimens (excluding edoxaban) appeared to lower the bleeding risk. Due to the fact that the above-mentioned trials were under-powered to assess the risk of thrombosis, it is still not known whether dual therapy (a NOAC plus a P2Y12 inhibitor) can sufficiently protect against myocardial infarction or stent thrombosis. Furthermore, the above-mentioned studies did not have a sufficient number of cases to obtain conclusive data regarding the safety of combined use of prasugrel or ticagrelor with P2Y12 inhibitors in dual or triple regimens. Hence, the safety of carrying out a PCI in NVAF patients on a NOAC needs to be further assessed in future prospective studies with large cohorts (Steffel et al., 2018).

## Critical Discussions

It is becoming increasingly clear that AF does not always act as a source of emboli. Using cardiac pacemakers and implantable cardioverter-defibrillators that store electrocardiogram (ECG) information for months, it was shown that emboli in many patients occur without AF (Healey et al., 2012; Brambatti et al., 2014). Hence, the current thinking is that AF may just be a marker of vascular disease but not the main cause of emboli.

Ethnic differences between Asians and non-Asians may also influence the optimal dosing of anticoagulants. Due to genetic differences or various eating habits, Asian populations, at least Japanese and Taiwanese populations, have lower levels of AF compared to Americans. This leads to clinical diversity in treatment approaches. For instance, acetylsalicylic acid is not useful for preventing AF complications in Japanese populations.

In addition, drug-drug interactions require further appraisal. The pharmacokinetic properties of NOACs may be a significant factor. In contrast to earlier claims by the industry that metabolism and the renal transport system play no clinically relevant role in NOACs efficacy and safety, there is now experimental evidence in cell culture and animal models, but more importantly in humans, that CYP450 enzymes and P-glycoprotein (P-gp) can affect NOACs pharmacokinetics (**Supplementary Material, Table S2**). Some interactions with common cardiovascular drugs, such as amiodarone, dronedarone, verapamil, and diltiazem have been previously discussed (**Supplementary Material, Table S3**) (Wiggins et al., 2020). Moreover, Chinese and Japanese patients seem to have alterations in CYP expression and function. Unfortunately, how these interactions affect the efficacy and safety of NOACs remains largely unknown. In summary, physicians should pay careful attention to patient ethnicity to determine who could be poor or rapid metabolizers (CYP450 system) and who may encounter unfavorable drug interactions and increased incidence of bleeding.

## Conclusion

NOACs have been approved to treat NVAF patients to diminish the risk of IS and SE in various countries due to their efficacy and safety profiles, which are either comparable or superior to those of the conventional treatment of warfarin, as presented in real-life registries and RCTs. First, nearly all landmark NOACs trials have excluded patients with a CrCl of <30 ml/min (with the exception of come patients on apixaban with CrCl 25–30 ml/min). However, the anticoagulant decision should be individualized, using a multidisciplinary approach based on a participant’s preferences. Second, even though there has been an increase in the use of NOACs, a certain proportion of patients remains on warfarin due to the high price of NOACs. Third, NOACs prescription and availability are regulated differently across countries. This is due to differences in inclusion criteria for RCTs. Fourth, NOACs either have not been researched or have demonstrated unfavorable results in patients with mechanical prosthetic heart valves or moderate to severe mitral stenosis (usually of rheumatic origin), in which circumstances warfarin continues to be the main treatment. Finally, despite the fact that NOACs possess relatively small drug interactions, physicians should carefully appraise the pharmacokinetic influences of accompanying medications (CYP and/or P-glycoprotein inducers or inhibitors) and comorbidities when prescribing NOACs.

Thus, this article serves as a guide outlining the suggested NOACs dosages for NVAF patients, and an overview on NOACs performance in AF patients with particular clinical characteristics. Our conclusions are based on dose adjustment recommendations in the literature, with the goal of presenting a simple and workable strategy for clinicians to choose appropriate NOACs.

## Ethics Statement

Ethical review and approval were not required for the study on human participants in accordance with the local legislation and institutional requirements. Written informed consent for participation was not required for this study in accordance with the national legislation and the institutional requirements.

## Author Contributions

SZ and PM raised the concept and design of the research. SZ wrote the manuscript. XH, JC, HC and SD revised the article for important details. All authors contributed to the article and approved the submitted version.

## Conflict of Interest

The authors declare that the research was conducted in the absence of any commercial or financial relationships that could be construed as a potential conflict of interest.

## Abbreviations

NOAC, non-vitamin K antagonist oral anticoagulants; NVAF, non-valvular atrial fibrillation; GIB, gastrointestinal bleeding; AF, atrial fibrillation; SE, systemic embolism; IS, ischemic stroke; VKAs, vitamin K antagonists; DOACs, direct oral anticoagulants; ICH, intracranial hemorrhage; SPAF, stroke prevention in atrial fibrillation; RCTs, randomized controlled trials; FDA, the US Food and Drug Administration; EMA, the European Medicines Agency; NMDA, the Chinese National Medical Products Administration; ICB, intracranial bleeding; ISTH, the International Society on Thrombosis and Haemostasis; EHRA, the European Heart Rhythm Association; CKD, chronic kidney disease; CrCl, creatinine clearance; ESKD, end-stage kidney disease; CAD, coronary artery disease; ACS, acute coronary syndrome; PCI, percutaneous coronary intervention; OAC, oral anticoagulation; DAPT, dual antiplatelet therapy; CRNM, clinically relevant non-major; P-gp, P-glycoprotein; ECG, electrocardiogram.
