# Supplementary material for: Appropriate Dosing Regimens of Non-Vitamin K Antagonist Oral Anticoagulants for Treatment of Patients With Non-Valvular Atrial Fibrillation: An Evidence-Based Consideration
Source: Front Pharmacol. 2020 Aug 20;11:1293. doi: 10.3389/fphar.2020.01293 (PMC7468491; doi:10.3389/fphar.2020.01293)
Supplement: Supplementary file 1 [file DataSheet_1.doc]

**Supplementary material**

**Table S1.** Approved dosing regimens for the NOACs package inserts for SPAF in the US, Europe, and Mainland China

| Dabigatran | FDA (US) (Revised: 03/2018) | EMA (Europe) (Revised: 01/2018) | NMDA (Mainland China) (Revised: 09/2019) |
| --- | --- | --- | --- |
|  | •75 mg twice daily:  -CrCl 30 – 50 mL/min with concomitant use of the P-gp inhibitor (dronedarone or systemic ketoconazole)  -CrCl 15 – 30 ml/min •150 mg twice daily:  -CrCl >30 ml/min •Dosing recommendations cannot be provided:  -CrCl <15 ml/min or on dialysis •Avoid co-administration  -CrCl <30 ml/min with concomitant use of P-gp inhibitors | •150 mg twice daily  •110 mg twice daily, if:  -age ≥80 years  -concomitant verapamil •Daily dose of 300 mg or 220 mg according to an individual evaluation of the thromboembolic risk and bleeding risk:  -age 75–80 years  -moderate renal impairment (CrCl 30–50 ml/min)  -gastritis, esophagitis or gastroesophageal reflex  -other increased breeding risk ^a^ | |
| Rivaroxaban | FDA (US) (Revised: 08/2019) | EMA (Europe) (Revised: 07/2018) | NMDA (Mainland China) (Revised: 12/2018) |
|  | •20 mg once daily with the evening meal:  -CrCl >50 ml/min •15 mg once daily with the evening meal:   -CrCl ≤50 ml/min | •20 mg once daily with food:  -CrCl ≥50 ml/min  •15 mg once daily with food:  -CrCl 15–49 ml/min | |
| Apixaban | FDA (US) (Revised: 06/2019) | EMA (Europe) (Revised: 05/2018) | NMDA (Mainland China) (Revised: 01/2019) |
|  | •5 mg twice daily •2.5 mg twice daily with at least two of the following characteristics:  -body weight ≤60 kg  -age ≥80 years  -serum creatinine ≥1.5 mg/dl (133μmol/L) | | Not approved |
| Edoxaban | FDA (US) (Revised: 08/2019) | EMA (Europe) (Revised: 06/2015) | NMDA (Mainland China) (Revised: 12/2018) |
|  | • 60 mg once daily • 30 mg once daily:  -body weight ≤60 kg  -CrCl 15 – 50 ml/min  -concomitant use of specific P-gp inhibitors (verapamil, quinidine, dronedarone) • Edoxaban should not be used in patients with CrCl >95 ml/min due to an increased risk of ischemic stroke compared to warfarin | • 60 mg once daily • 30 mg once daily with one or more of the following clinical factors:  -CrCl 15–50 ml/min  -body weight ≤60 kg  -concomitant use of the following P-gp inhibitors: ciclosporin, erythromycin, dronedarone, or ketoconazole  • Edoxaban should only be used in NVAF patients with high CrCl after a careful evaluation of the individual thromboembolic and bleeding risk | |

a: Other increased bleeding risks include: Strong P-gp inhibitors; mild to moderate P-gp inhibitor co-medications (e.g. quinidine, verapamil, ticagrelor, and amiodarone); low body weight (< 50 kg); acetylsalicylic acid (ASA) and other platelet aggregation inhibitors (e.g. clopidogrel); selective serotonin norepinephrine re-uptake inhibitors (SNRIs), selective serotonin re-uptake inhibitors (SSRIs), and non-steroidal anti-inflammatory drugs (NSAID); or other medicinal products that may impair hemostasis; functional platelet defects or thrombocytopenia; major trauma and recent biopsy; bacterial endocarditis.

SPAF = stroke prevention in atrial fibrillation; NOAC = non-vitamin K antagonist oral anticoagulant; FDA = Food and Drug Administration; EMA = European Medicines Agency; NMDA = National Medical Products Administration; P-gp = P-glycoprotein; CrCl = creatinine clearance.

**Table S2.** Pharmacologic properties of NOACs

| NOAC | Dabigatran | Rivaroxaban | Apixaban | Edoxaban |
| --- | --- | --- | --- | --- |
| Bioavailability, % | 3-7 | 66 without food; 80-100 with food | 50 | 62 |
| Prodrug | Yes | No | No | No |
| Plasma protein binding, % | 34-35 | 92-95 | 87 | 55 |
| Distribution volume, L | 60-70 | 50 | 21 | 107 |
| Time to C_max_, h | 0.5-2 | 2-4 | 3-4 | 1-2 |
| Elimination half-life, h | 12-17 | Young: 5-9;  Elderly: 11-13 | 12 | 10-14 |
| Renal excretion, % | 80 | 35 | 27 | 50 |
| P-gp substrate | Yes | Yes | Yes | Yes |
| CYP 3A4 substrate | No | Yes (≈18%) | Yes (≈25%) | No (< 4%) |
| Asian ethnicity, % | +25 | No effect | No effect | No effect |

NOAC = non-vitamin K antagonist oral anticoagulant.

**Table S3.** Recommendation summaries for interactions with NOACs and cardiovascular agents

| **Cardiovascular Agent** | **NOACs** | **Recommendation Summaries** |
| --- | --- | --- |
| Amiodarone | Dabigatran | Combination considered safe if CrCl >50 ml/min.  Avoid combination if CrCl <50 ml/min for VTE and <30 ml/min for NVAF. |
|  | Rivaroxaban | Avoid use if CrCl <80 ml/min. |
|  | Apixaban | Combination is considered safe. |
|  | Edoxaban | Combination is considered safe. |
| Dronedarone | Dabigatran | Administer 2 h before dronedarone. Reduce dose to 75 mg twice daily for CrCl 30–50 ml/min. Avoid use if CrCl <30 ml/min. |
|  | Rivaroxaban | Avoid combination if CrCl <80 ml/min |
|  | Apixaban | Combination is considered acceptable |
|  | Edoxaban | Reduce dose of edoxaban by 50% |
| Verapamil | Dabigatran | Avoid use if CrCl <30 ml/min for NVAF and <50 ml/min for VTE. |
|  | Rivaroxaban | Avoid combination when CrCl is <80 ml/min. |
|  | Apixaban | Combination is considered safe. |
|  | Edoxaban | Combination is considered safe. |
| Diltiazem | Dabigatran | Combination is considered safe. |
|  | Rivaroxaban | Avoid use if CrCl <80 ml/min. |
|  | Apixaban | Combination is considered safe. |
|  | Edoxaban | Combination is considered safe. |

NOAC = non-vitamin K antagonist oral anticoagulant; CrCl = creatinine clearance. VTE = venous thromboembolism; NVAF = non-valvular atrial fibrillation.

The data summarized in this table are from .

**Figure S1. Coagulation cascade. Targets of anticoagulant agents.**


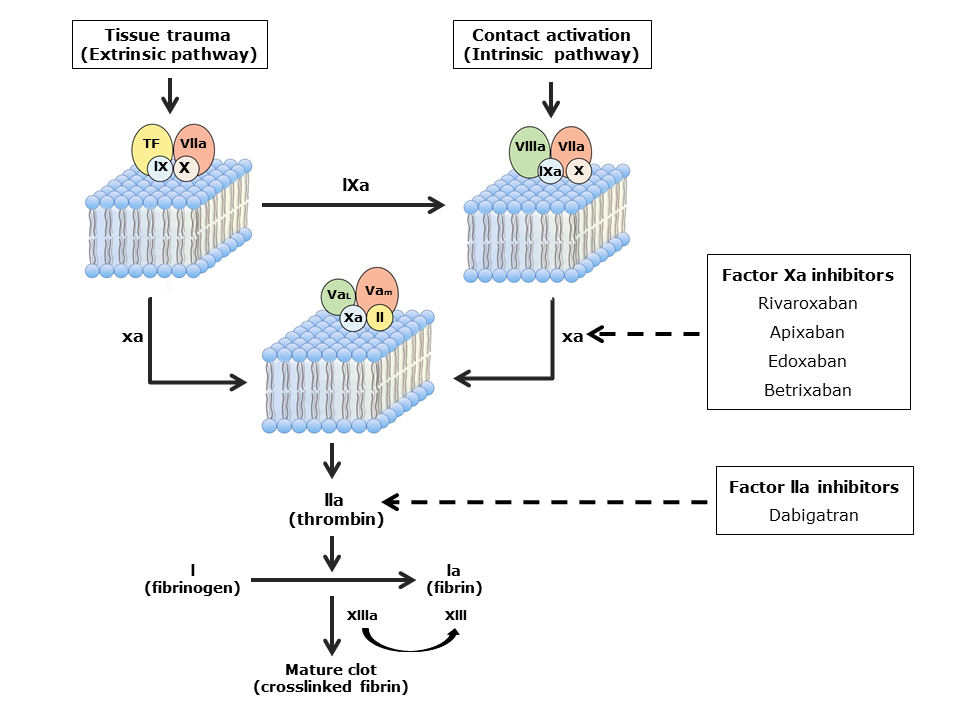


**Figure S2. Major hemorrhage in the ≥ 75 years subgroups based on landmark phase III AF RCTs.**


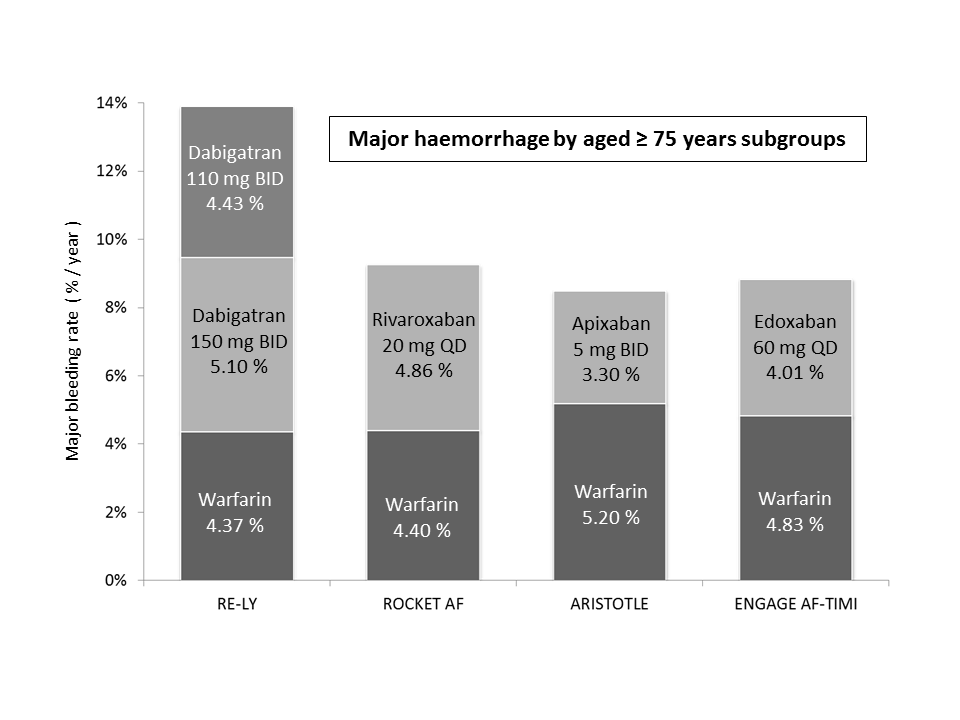


AF = atrial fibrillation; RCTs = randomized controlled trials.

The data summarized in this figure are from the following studies: Connolly, 2009; Patel, 2011; Granger, 2011; and Giugliano, 2013.

**Figure S3. Major GIB rates in patients with AF treated with NOACs versus warfarin.**


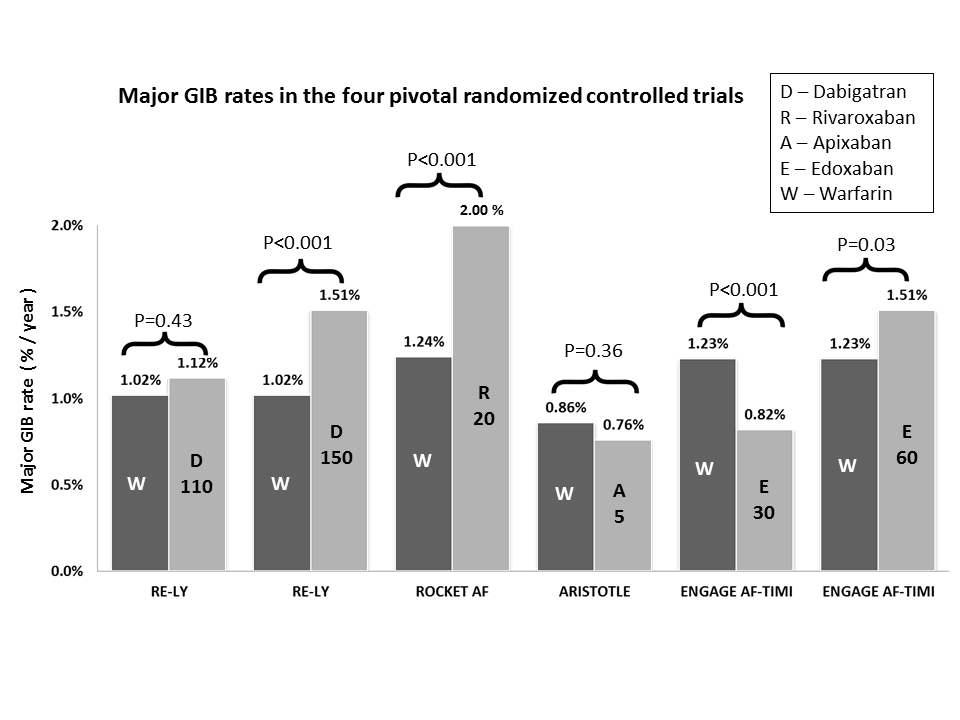


GIB = gastrointestinal bleeding; NOAC = non-vitamin K antagonist oral anticoagulants; AF = atrial fibrillation.

The data summarized in this figure are from the following studies: Connolly, 2009; Patel, 2011; Granger, 2011; and Giugliano, 2013.
